# Thoracic applications of photon-counting CT: where are we after 3 years of clinical implementation?

**DOI:** 10.1093/bjr/tqaf026

**Published:** 2025-03-07

**Authors:** Martine Remy-Jardin, Thomas Flohr, Jacques Remy

**Affiliations:** Thoracic Imaging, IMALLIANCE-Haut-de-France, F-59300 Valenciennes, France; Department of Radiology, Radboud University Medical Centre, 6500 HB Nijmegen, The Netherlands; Department of Computed Tomography Research & Development, Siemens Healthineers AG, 91301 Forchheim, Germany; Department of Radiology and Nuclear Medicine, Maastricht University Medical Centre, 6202 AZ Maastricht, The Netherlands; Department of Radiology, Valenciennes Regional Hospital, F-59300 Valenciennes, France

**Keywords:** photon-counting CT, spectral imaging, radiation dose, CT angiography

## Abstract

Photon-counting CT has now entered the field of clinical practice, raising expectations on the advantages that could be derived for patient management. Two main directions are under scrutinity for the medical community at large. At the present time, most attention is directed towards the confirmation of the expected improvement in image quality and the evaluation of its consequences in terms of decision-making. In parallel, new perspectives in the field of functional imaging as well as for spectral imaging are topics of active research that have not been translated in clinical practice. This review article provides an update on the current use of this technology, based on the last 3 years of clinical investigations. Early clinical experience is summarized, focusing on adult respiratory indications.

## Introduction

After a decade of preclinical evaluation, photon-counting-detector CT (PCD-CT) has started a new phase of evaluation that takes place in the conditions of routine clinical practice. This has become possible with the installation of the first commercial PCD-CT equipments that has triggered the publication of a considerable number of articles over the last 3 years, including detailed review articles.^1^^,^^2^ In the present review article, our objective is to prolong our knowledge of the clinical benefits of this new technology, foreseen during the period of transition from prototypes to clinical routine.^3^ The current period is very active, with numerous technological advances proposed on the systems clinically investigated while all major manufacturers are currently developing PCD-CT systems. In this article focusing on chest imaging in adults, our goal is to gather the main results of prime-time investigations in the two main fields of clinical application of PCD-CT, namely ultra-high-resolution (UHR) and CT angiography, both taking advantages of radiation dose reduction.

## Technical features of PCD-CT

PCDs convert the incident X-ray quanta directly into electrical pulses with a pulse height proportional to the X-ray energy. The pulses are counted as soon as they exceed a threshold value. By applying several different thresholds simultaneously, PCDs can routinely provide spectral CT data in different energy bins. Unlike conventional energy integration scintillation detectors (EIDs), PCDs are insensitive to electronic background noise. Therefore, CT images at low X-ray flux show better image quality with less noise. PCDs can be much more finely structured than EIDs to improve spatial resolution, as no separating layers between the individual detector elements are required to prevent optical crosstalk. While lung imaging with conventional CTs is limited to a resolution of about 0.4 mm, PCD-CTs can provide a resolution better than 0.2 mm for UHR lung scanning.

A commercial dual-source PCD-CT (NAEOTOM Alpha, Siemens Healthineers AG, Forchheim, Germany) enables either standard-resolution scans at 144 × 0.4 mm detector collimation or UHR scans at 120 × 0.2 mm collimation, with a maximum in-plane resolution of 0.208 mm (standard) and 0.125 mm (UHR). Spectral data are available for both standard and UHR scans, but only in standard resolution (smallest slice thickness 0.4 mm). The data from four energy bins are combined into two spectral channels for established dual-energy CT applications. The scanner implements an approach using 120 or 140 kV tube voltage for all routine protocols. At standard resolution, the primary result of each scan is a virtual monoenergetic image (VMI) calculated from the data of the two spectral channels. In a VMI, the attenuation of iodine contrast medium is reproduced as if the CT data had been acquired with a monoenergetic X-ray beam of the corresponding energy. The VMI energy (keV-level) is customized to the respective clinical question, which is, eg, 70 keV for non-contrast scans, or 55 keV for CT angiography. The iodine contrast-to-noise ratio (CNR) of the VMIs is improved by refined algorithms such that it increases with decreasing keV similarly as if the images were acquired with lower tube voltage (kVp). In addition, contrast-enhanced lung scans routinely provide iodine maps of the lung parenchyma, which show lung perfusion, and virtual non-contrast (VNC) images, in which the iodine has been removed.

## What has already been achieved with UHR in lung imaging?

The use of smaller detector pixels on PCD-CT on PCD-CT enables UHR imaging at 0.2 mm slice width and is synonymous of improved visualization of subtle changes and/or depiction of anatomical structures compared with high-resolution CT examinations obtained with EID-CT. However, the simultaneously increased in-plane resolution may make it necessary to use a 1024-image matrix instead of the usual 512-image matrix. The large number of 0.2 mm images with a 1024 matrix in a lung scan can pose challenges for data archiving. In addition, higher resolution in CT always means higher objective image noise.

Using research PCD-CT scanners, preliminary investigations confirmed the expected results of the UHR scanning mode reported in experimental studies, namely a sharper airway wall delineation and visualization of higher-order bronchi.^4–6^ Using a commercial dual-source PCD-CT scanner, Gaillandre et al^7^ demonstrated that radiologists could visualize up to 13th-order bronchial divisions in daily routine. The sharpness of fissures was also found to be superior with the UHR mode, raising expectations on the possibility of more precise depiction of subtle parenchymal changes. It is noteworthy that early clinical experiences reported that improved resolution could be achieved while substantially reducing the radiation dose of chest CT examinations. It is important to underline that EID-CT relies on comb or grid filters to narrow the detector aperture. Since PCD-CT does not require these filters, UHR scans can be realized without dose penalty. As a result, direct comparisons between dose-matched UHR and standard-resolution PCD-CT scans are now feasible. Huflage et al^8^ and Martinez et al^9^ were able to show that a considerable noise reduction can be achieved when UHR datasets are reconstructed with the same sharpness as standard-resolution scans (ie, the so-called “small pixel effect”). This phenomenon appears particularly promising for ultra-low-dose lung imaging.

### UHR in ILDs

**Figure 1.**
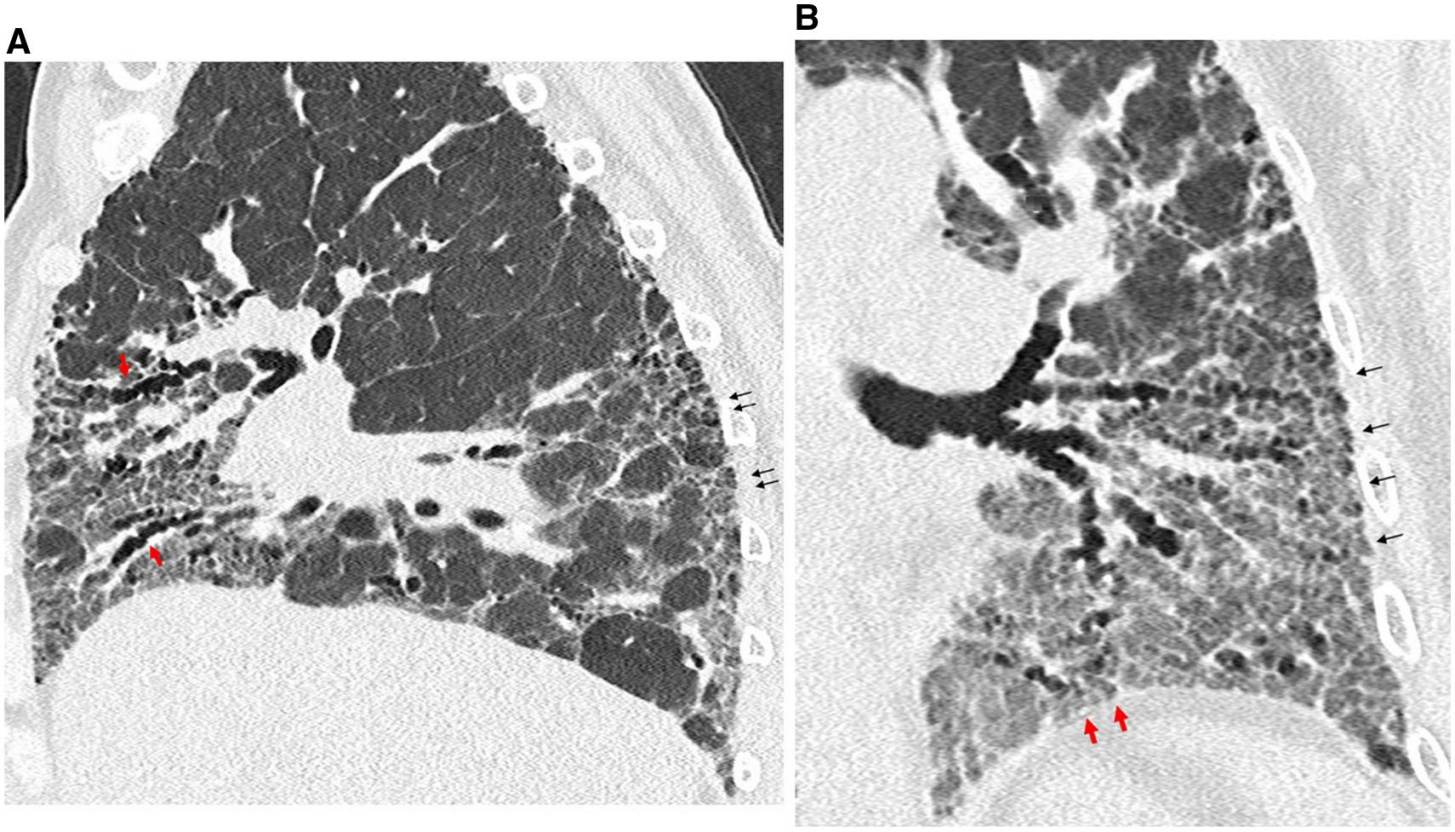
Example of ultra-high-resolution (UHR) images in a 67-year-old male patient with fibrotic hypersensitivity pneumonitis. Both images are generated from an acquisition obtained with a 0.2 mm collimation, reconstructed with 0.6-mm section thickness, 512 matrix. Saggital (A) and coronal (B) images showing precise delineation of peripheral traction bronchiectasis (red arrows) and depiction of intralobular reticulation in the posterior and lateral parts of both lungs (black arrows).

**Figure 2.**
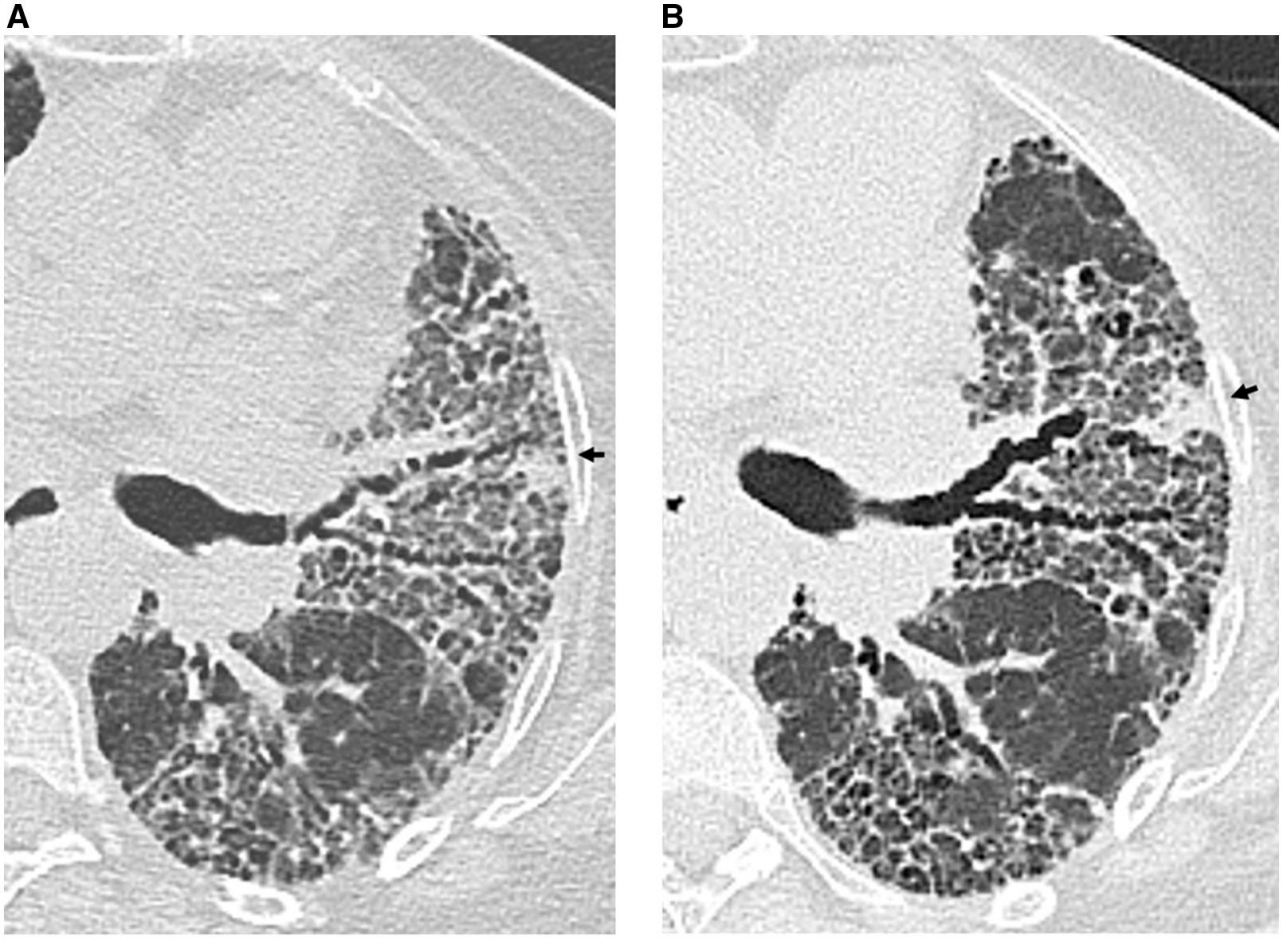
Example of a 72-year-old male patient with fibrotic non-specific interstitial pneumonia (NSIP) scanned 1 year apart. (A) Axial image from an energy-integrating-detector CT (third generation dual-source CT system) (0.75 mm section thickness) obtained at the level of the left upper lobe bronchus, showing CT features of bilateral fibrotic lung disease. (B) Axial PCD-CT image, obtained at the same anatomical level as shown in A 1 year later (0.2 mm collimation, reconstructed with 0.6-mm section thickness, 512 matrix). Compared to A, note the modifications in the patterns of lung involvement in the apical segments of both lower lobes, providing objective clues for disease progression. Note the considerable increase in size of the left upper lobe nodule (*arrow on both images*), highly suspect of malignancy developing in an area of lung fibrosis.

Based on these results, the UHR scanning mode was logically investigated in interstitial lung diseases (ILDs), relying on comparative analyses between UHR-PCD-CT and high-resolution EID-CT examinations. If all studies confirm the sharper delineation of CT features of ILD with PCD-CT^7^^,^^10–13^ (Figures 1 and 2), the clinical impact of the improved spatial resolution remains debated, in the background of efforts aimed at depicting early fibrosis at a time when antifibrotic drugs could hamper development of irreversible structural damage of the lung parenchyma. In a series of 61 patients with rheumatoid arthritis, Marton et al^10^ showed more detailed information on ILD but the pattern of lung parenchymal involvement did not differ between the two scanning modes. Similar conclusions were drawn by Van Ballaer et al^12^ who compared the clinical image quality and perceived impact on diagnostic interpretation of chest CT findings. Despite superior image quality, the readers perceived no significant impact on the diagnostic interpretation of the studied lung structures and abnormalities. In a large cohort of 112 patients, Gaillandre et al^7^ reported an overall improvement in image quality of PCD-CT examinations with a clinically relevant impact in terms of ILD categorization in four patients. The detection of traction bronchiolectasis on PCD-CT UHR images that were not observed in high-resolution EID-CT images led to the reclassification of non-fibrotic ILD to fibrotic ILD. Investigating a cohort of 20 patients with post COVID-19 persisting symptoms, Prayer et al^11^ reported that fine reticulations were visible in PCD-CT but described as GGO at EID-CT. They underlined that this finding corroborates observations by Inoue et al^14^ who reported several instances of GGO at EID-CT that represented reticulations at same-day PCD-CT in patients suspected of having ILD. The unmasking of fine reticular opacities within areas of GGO at PCD-CT may help identify patients at risk for developing irreversible fibrosis. To our knowledge, a single study studied correlations between PCD-CT findings and pulmonary function tests.^10^ These authors reported a mild negative correlation between total scores of ILD extent and diffusion capacity for carbon monoxyde.

**Figure 3.**
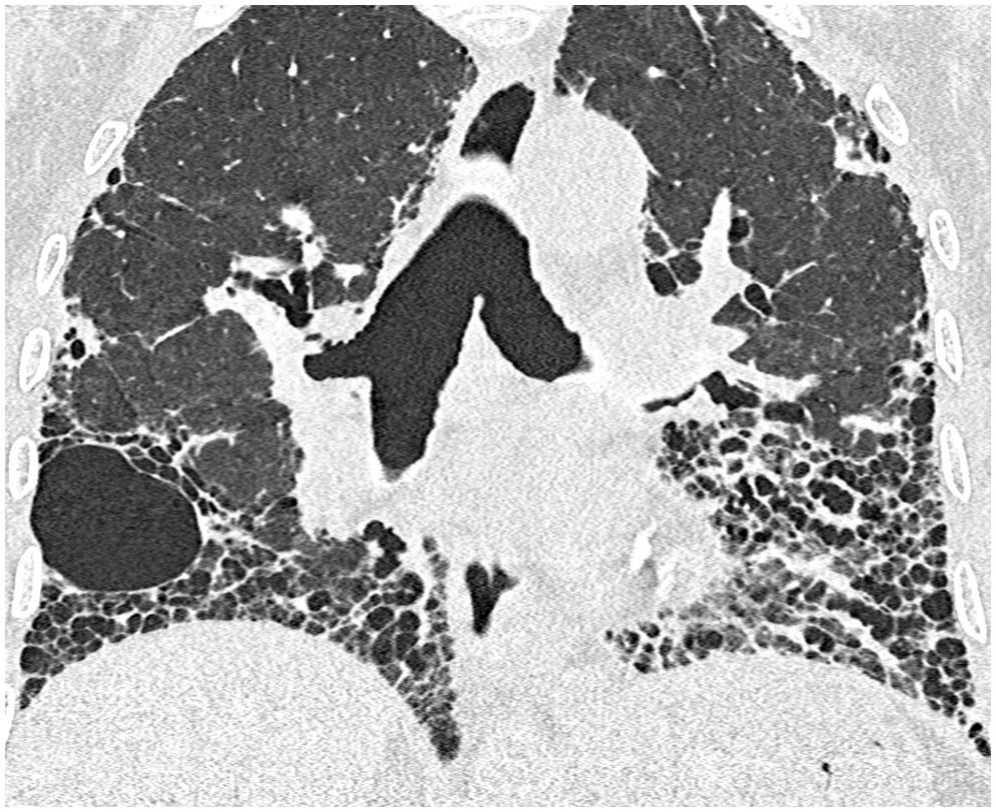
Example of a 69-year-old male patient with idiopathic pulmonary fibrosis. Coronal PCD-CT image (0.2 mm collimation, reconstructed with 1-mm section thickness, 512 matrix) obtained at the level of tracheal bifurcation, illustrating the sharp delineation of honeycombing in both lungs as well as cyst walls in the right middle lobe. Note the lack of motion artefacts around the cardiac cavities, especially in the left lung.

In parallel to the clinical evaluations, numerous technical parameters are tested to propose optimized PCD-CT scanning protocols, including kernels and slice thicknesses for routine lung examinations,^15^ optimal strength levels of novel iterative reconstruction algorithms for low-dose, UHR images of PCD-CT examinations.^16^ In the context of systemic sclerosis, Jungblut et al^17^ reported that a radiation dose reduction of 66% was feasible with PCD-CT in comparison with EID-CT, without penalty in image quality and diagnostic performance for the evaluation of ILD. In the specific context of low-dose UHR-PCD-CT of the lungs, Graafen et al^18^ suggest that the optimal reconstruction protocol includes a slice thickness of 0.4 mm with the highest QIR level. Scanning patients with a high pitch ensures considerable reduction in motion artefacts (Figure 3).

### UHR outside the field of ILDs

In an experimental study, Dunning et al^19^ demonstrated that high-spatial-resolution PCT-CT improved accuracy of pulmonary nodule volumetry and shape characterization. Based on PCD-CT radiological-histological correlation in cadaveric human lung nodules and airways, Hata et al^20^ have recently shown that PCD-CT visualized small nodules and airways better than EID-CT, detecting submillimeter nodules and airways. The median size of barely detectable nodules was 604 µm (vs 837 µm) and the size of barely detectable airways was 601 µm (vs 1210 µm). Fully automatized artificial intelligence (AI)-aided nodule detection and volumetry was found to be feasible with PCD-CT.^21^ In this study based on the evaluation of an anthropomorphic phantom containing nodules of various sizes, the authors tested the applicability of a commercially available EID-trained AI-based computer-aided detection (CAD) system in PCD-CT. Considering subjective and objective IQ, radiation dose, and performance in pulmonary nodule evaluation, PCD-CT using the “high-resolution” mode (120 × 0.2 mm) at the lowest radiation dose (CTDI_vol_ of 0.41 mGy) delivered the best trade-off between radiation dose reduction and nodule performance. Using the “standard” acquisition mode (140 × 0.4 mm), they observed an increase in sensitivity at lower doses but, at the same time, the rate of the false-positive finings rose to unacceptably high levels, making the algorithms' overall performance worse at lower radiation dose levels. They subsequently concluded that PCD-CT poses a new challenge for AI-based CAD systems, and additional training and recalibration on PCD-CT datasets might be needed before CAD systems can be reliably used in PCD-CT datasets.

Lung density quantification is also of particular interest, especially in the field of chronic obstructive pulmonary disease. A phantom study comparing PCD-CT to EID-CT showed that PCD-CT allowed more accurate quantification of biomarkers of emphysema, such as the low-attenuation areas below −950 HU and the CT attenuation value at the 15th percentile of the lung CT histogram.^22^ More recently, Sotudeh-Paima et al^23^ confirmed superior performance in density quantifications with a clinical PCD-CT scanner, suggesting new options for more objective assessment of respiratory conditions. Graafen et al also showed that PCD-CT has the potential to reveal discreet anatomical alterations that enable earlier and more precise detection of lung diseases. They suggest that this feature might be accessible through improved radiomics features with better cluster separation.^24–26^ Depending on the scanning conditions of COPD patients, it is worth emphasizing additional results on the quantification of emphysema with PCD-CT. In patients undergoing a PCD-CT angiographic examination using a multi-energy mode, emphysema quantification was reported to be feasible and accurate using virtual non-contrast images compared to true non-contrast images.^27^ Estimation of the severity of emphysema has also been tested on PCD-CT scans performed at low-dose and with X-ray equivalent doses.^28^ The authors found that the severity of emphysema was reliably estimated by visual scoring on PCD-CT scans performed with X-ray equivalent doses. A deep-learning algorithm demonstrated good agreement and strong correlation with the visual scoring method on low-dose scans. However, both the deep-learning and low-attenuation volume algorithms overestimated emphysema extent on X-ray dose PCD-CT scans. These pioneering studies open the field of new approaches in the quantitative evaluation of COPD patients. A potential drawback of UHR imaging has to be considered, namely the raw data file sizes and reconstruction times. Since the demand for UHR scans will undoubtedly increase in the future, it is mandatory for radiologists to investigate new options in terms of reconstruction protocols and workflows to cope with the increasing data volumes.

## PCD-CT angiographic examinations of the chest

### Chest CT angiography in daily practice

**Figure 4.**
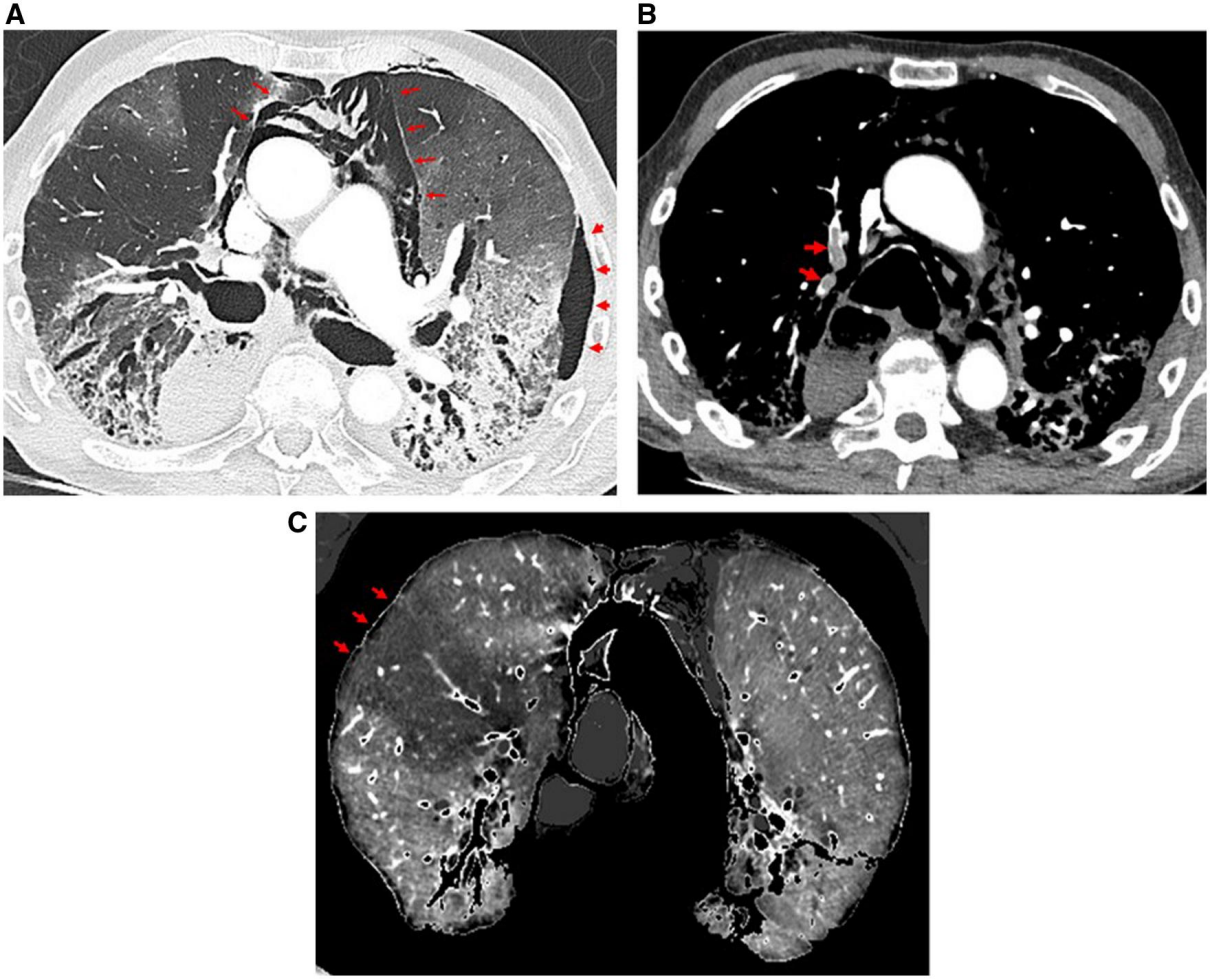
Example of a 73-year-old male patient with acute exacerbation in the context of idiopathic pulmonary fibrosis (IPF). Chest CT angiographic examination obtained with PCD-CT (0.4 mm collimation; pitch of 1; 70 keV lung and mediastinal reconstructions). (A) Axial PCD-CT lung image (1-mm section thickness) at the level of the pulmonary trunk, illustrating the presence of bilateral ground-glass opacities and the concurrent presence of a left pneumothorax (small arrows) and pneumomediastinum (long arrows) in the context of fibrotic lung disease. (B) Axial PCD-CT mediastinal image (1-mm section thickness) obtained 2 cm above A, showing endoluminal filling defects in the anterior and apical segmental arteries of the right upper lobe (arrows). Note the sharp delineation of endoluminal clots in both arterial sections. (C) Axial PCD-CT lung perfusion image (1-mm section thickness) obtained at the level of the aortic arch, showing a focal PE-type perfusion defect in the right upper lobe (arrows).

The benefits of PCD-CT for CT angiographic examinations cannot be better illustrated than in the context of acute pulmonary embolism (PE) (Figure 4). In a large cohort of patients, Remy-Jardin et al^29^ compared the conditions of acute PE detection and image quality when PCD-CT replaces EID-CT in daily routine. As the speed of data acquisition is a key parameter to generate high-quality images, the mean duration of data acquisition in less than 1 s with PCD-CT accounted for the possibility of examining all patient categories with spectral imaging, whereas it was only possible for 66.5% of patients with EID-CT. They also reported that 89% of examinations were devoid of artefacts vs 28.6% when scanning patients with dual-source, dual-energy CT, leading Andersen choose on purpose the following title for his editorial “When radiologists don’t have to choose between image quality or motion artifacts”.^30^ Since this preliminary experience limited to the use of a pitch of 1.5, high-quality morphologic imaging with higher pitch values has been reported in the context of acute PE.^31–33^ Using a pitch value of 3.2, it becomes logically possible to scan patients with a free-breathing technique.^34^ Compared to EID-CT, this scanning technique also allowed for superior objective and subjective image quality.

Two additional advantages of PCD-CT angiographic examinations have to be underlined. First, PCD-CT ensures considerable dose reduction compared to EID-CT.^29^^,^^31^^,^^34^ The second advantage is the potential of PCD-CT for contrast medium reduction. The image quality of an ultra-low contrast medium protocol was recently tested in the context of clinical suspicion of acute PE, relying on the administration of 25 mL of a 35% contrast material that was compared to the results achieved with 50 mL of the same contrast agent using an EID-CT protocol. Whereas the authors reported no “non-diagnostic” examinations in either group, the objective image quality parameters were significantly higher in the EID-CT group, both in the polychromatic reconstructions and at 60 keV. These results were explained by major differences between scanning protocols, the PCD-CT scanning mode combining a 51% radiation dose reduction and the administration of a considerably lowered iodinated contrast volume compared to the EID-CT protocol. Despite these differences, the authors mentioned that the decrease in contrast-to-noise ratio between detector systems was acceptable in any case, especially when taking higher resolution, less motion artefacts, and the achieved severe dose reduction saving with the PCD system into account.

This protocol can be advantageous for patients with impaired renal function but it does not allow to generate perfusion maps.^31^ Diagnostic PCD-CT angiograms have also been reported after administration of 35 mL of a 30% contrast agent.^35^ Similar trends can be observed for PCD-CT angiographic examinations of the aorta. In a recent paper by Higashigaito et al,^36^ PCD-CTA of the aorta was associated with higher CNR, which could be translated into a low-volume contrast media protocol, demonstrating noninferior image quality compared with EID-CT at the same radiation dose.

As spectral imaging is available for each and every examination, it is interesting to question the quality of iodine maps of the lung parenchyma. Comparing PCD-CT and EID-CT using similar injection protocols, Remy-Jardin et al^37^ showed that high-quality lung perfusion imaging can be generated from PCD-CT examinations with higher spatial resolution and reduced cardiac motion artefacts, with a mean reduction of 52% of the radiation dose. The improvement in the overall quality of perfusion images at lower radiation doses opens the door for wider applications of lung perfusion imaging in clinical practice. A new direction has recently been reported, using iodine maps to characterize parenchymal lung disease, improving the diagnostic confidence of radiologists in the characterization of parenchymal pathologies.^38^ High-quality perfusion maps should also be of great interest in the diagnostic approach of chronic thromboembolism, especially in the peripheral forms of the disease as already introduced with dual-energy EID-CT.^39^ Whatever using EID-CT or PCD-CT, one should keep in mind that the diagnosis of chronic obstruction of the pulmonary circulation with or without pulmonary hypertension cannot be assessed on iodine maps alone,^40^ the key diagnostic features remaining the identification of the vascular signs of the disease.

### Additional aspects of spectral imaging with PCD-CT

**Figure 5.**
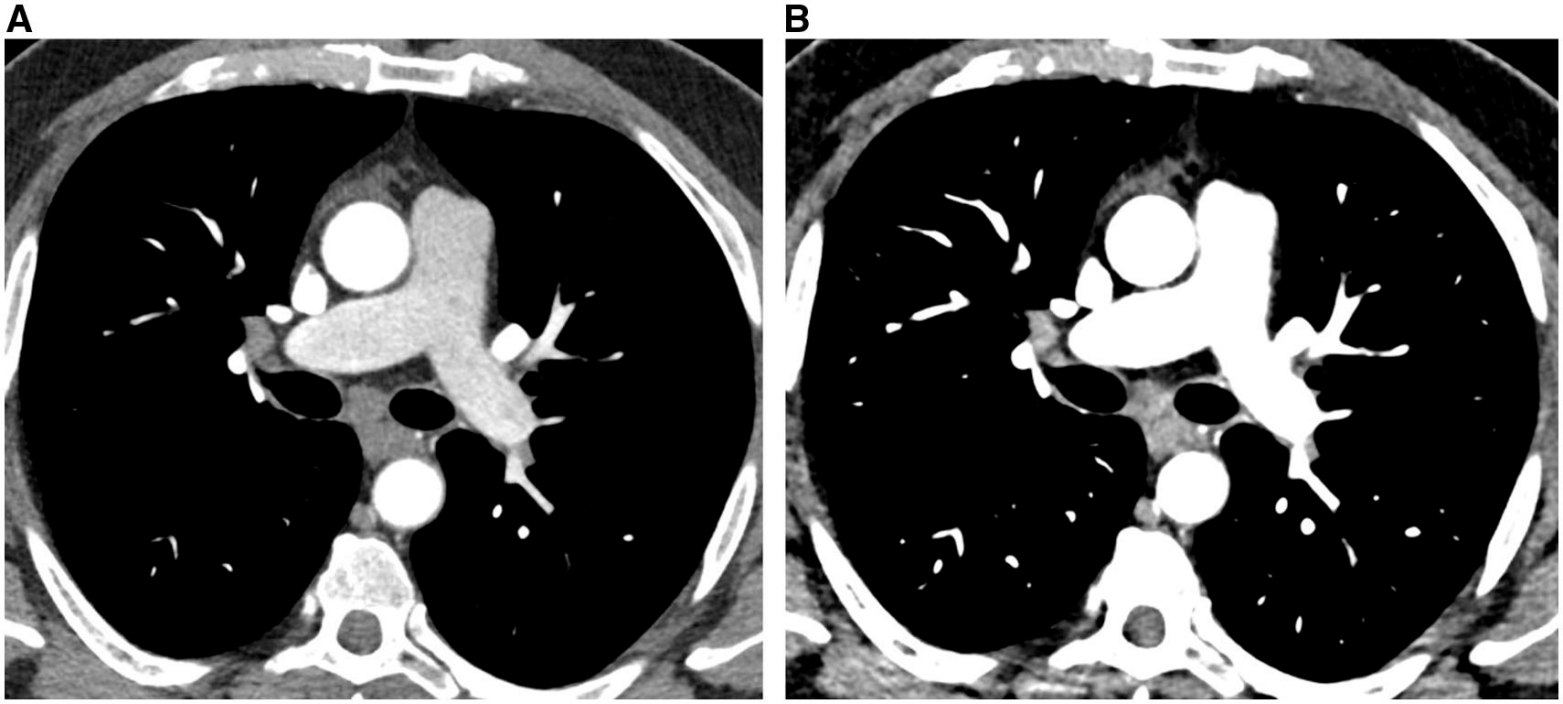
Example of a 45-year-old male patient referred for suspicion of acute pulmonary embolism. Chest CT angiographic examination obtained with PCD-CT (0.4 mm collimation; pitch of 1). (A) Axial PCD-CT mediastinal image reconstructed at 70 keV, showing suboptimal opacification of pulmonary arteries secondary to a Valsalva manoeuver. (B) Axial PCD-CT mediastinal image reconstructed at 40 keV. The low-energy reconstruction enables restoration of optimal enhancement of pulmonary arteries. The quality of vascular opacification enabled confident exclusion of acute pulmonary embolism down to the subsegmental level.

A reduction of acquisitions can be obtained by reconstruction of VNC images from a chest CT angiographic examination, participating in the radiation dose reduction. One study demonstrated the feasibility of quantifying emphysema on VNC images generated at clinical PCCT from arterial and portal phases with high accuracy compared with true non-contrast images.^27^ High-quality angiograms ensured by the availability of VMIs that can restore a diagnostic image quality by enhancing the degree of vascular opacification at low-keV level (Figure 5). Low-energy VMIs have recently been shown to be a useful adjunct to differentiate empyema and non-infected pleura.^41^

Beam-hardening artefacts secondary to the presence of metallic structures can be reduced using high-energy VMIs but this kind of artefact is not a major problem in chest imaging. More frequent is the problem of beam-hardening artefacts around systemic veins opacified with highly concentrated contrast agents. Similarly to the solutions proposed with dual-energy CT, one can consider reducing the iodine concentration of the administered contrast agent or acquire data sets at the time of recirculation. A field currently under investigation concerns the combination of ultra-high-spatial resolution of PCD-CT with spectral properties. In a recent study based on a phantom experiment, Mihailovic et al^42^ showed that PCD-CT with combined multi-energy and high-pitch mode facilitated simultaneous accurate iodine quantification and assessment of intravascular occlusion.

Among other expected improvements with PCD-CT, one should underline the options linked to the so-called k-edge imaging that could help discriminate between two contrast materials for lung ventilation and perfusion, as well as the development of new contrast media. While awaiting these new clinical applications, Scharm et al^43^ showed the possibility of simultaneously evaluating both pulmonary morphology, ventilation, vasculature, and parenchymal perfusion based on an inspiratory scan performed with PCD-CT followed by an expiratory scan after a 5 min waiting period.

## Low-dose and ultra-low dose PCD-CT: current results and perspectives

Most investigations started to compare the radiation dose of PCD-CT with that of up-to-date EID-CT. Reduced by 32%,^6^ this level of dose reduction of UHR examinations is in the range of that reported by Woeltjen et al^44^ who observed a 35.7% dose reduction compared to EID-CT scans. With PCCT, a radiation dose reduction of 66% compared with EID-CT was reported to be feasible, without penalty in image quality and diagnostic performance in the evaluation of systemic sclerosis.^17^ Similar results are achievable in other conditions such as pulmonary nodules.^19^ It is also interesting to underline that the improved resolution at lower radiation dose is also reported in obese patients.^13^

A better image quality at lower radiation dose with PCD-CT opens new horizons for further dose reduction in non-contrast UHR examinations. This is crucial for patients requiring frequent CT follow-up over long periods, such as patients investigated for incidental pulmonary nodules, ILDs and young patients in the context of oncology care. A recent study by Milos et al^45^ has shown that it is possible to use ultra-low-dose PCD-CT for routine surveillance of patients after lung transplant. A similar approach has been reported in early cystic fibrosis lung disease.^46^ These authors approach the paradigm of using chest CT at a comparable dose to that used in standard chest radiography. A broad applicability of ultra-low-dose chest PCD-CT examinations remains to be demonstrated, keeping in mind the results achievable with EID-CT. A systematic review analysis showed that low-dose CT had high diagnostic accuracy in detection of honeycombing and bronchiectasis and ultra-low-dose CT a high diagnostic accuracy for pneumothorax, consolidations and ground-glass-opacities while there were varying sensitivities for nodules and emphysema.^47^ Dettmer et al^48^ evaluated the clinical yield of an ultra-low-dose CT PCD-CT protocol with the radiation dose comparable to the dose of a CXR in patients with suspicion of pneumonia. They showed that this approach could become relevant in selected patients with respiratory infections and unclear chest X-ray or clinical presentation as well as in patients under immunosuppression.

In the context of lung cancer screening, low-dose UHR scanning protocols with PCD-CT raise great interest owing to the possibility to minimize the radiation dose for potentially healthy individuals while ensuring satisfactory image quality. Inoue et al^49^ evaluated the diagnostic quality of low-dose PCD-CT in patients undergoing lung cancer screening compared with conventional EID-CT in a prospective multireader study. With similar acquisition and reconstruction settings, PCD-CT demonstrated improved image quality and less noise despite lower radiation dose, with improved ability to depict pulmonary emphysema and lung nodule borders. It appears important to mention the advantages of spectral shaping via tin prefiltration, especially in low-dose lung imaging.

## Conclusion

Chest CT imaging is constantly evolving, and the radiological community has started a new era of investigations with PCD-CT in clinical routine. The current period reflects a great enthusiasm in academic centres where numerous fields of potential applications are explored while the costs of PCD-CT systems currently prevent wider implementations. Further scientific evidence is needed to define the impact in patient management and more technically oriented developments are necessary to handle the substantial increase in CT data volume.
